# Need for better reporting of trials with surrogate endpoints: SPIRIT|CONSORT-SURROGATE extensions

**DOI:** 10.1136/jech-2022-219294

**Published:** 2022-06-24

**Authors:** Oriana Ciani, Anthony Manyara, Rod S Taylor

**Affiliations:** 1 SDA Bocconi School of Management, Milano, Italy; 2 MRC/CSO Social and Public Health Sciences Unit, University of Glasgow, Glasgow, UK; 3 MRC/CSO Social and Public Health Sciences Unit & Robertson Centre for Biostatistics, Institute of Health and Well Being, University of Glasgow, Glasgow, UK

**Keywords:** EPIDEMIOLOGY, RANDOMIZED CONTROLLED TRIAL, STUDY DESIGN

Evidence for the effectiveness of health interventions should ideally come from randomised trials that assess a participant relevant final outcome (PRFO), such as health status or survival.^1 2^ However, such trials often require large sample sizes, long follow-up times and are resource intensive and costly.^2^ Surrogate endpoints or ‘surrogates’ have been used to improve trial efficiency by acting as a proxy and predictor for PRFOs.^3^ Over the last two decades, drug licensing in the USA and Europe has allowed the use of biomarkers (an objectively measured molecular, histologic, radiographic or physiologic characteristic) as surrogates in the approval of new therapies, for example, systolic blood pressure and/glycosylated haemoglobin (HbA1c) for cardiovascular death, HIV viral load for development of AIDS and tumour response for overall survival.^3 4^ However, it is important to recognise the application of surrogates in the wider setting of healthcare evaluation (including trials of public health, diagnostic, surgical, mental health, primary care, rehabilitation interventions) and the use of so-called intermediate outcomes (outcome on the causal path for PRFO that can be measured earlier and are predictive) as surrogates, for example, hospice enrolment for mortality with an intervention aimed at improving end of life care^5^; fruit and vegetable consumption for cardiovascular events for a behavioural intervention designed to improve cardiovascular risk.^6^

Despite their benefits, the use of surrogates in evaluation and regulatory approval of health interventions remains controversial. First, some therapies, approved based on surrogates, have failed to deliver improved PRFOs, and in some cases, cause more overall harm than good, treatment effects are often not all mediated through the surrogate–PRFO causal pathway.^7^ An example is the diabetes therapy rosiglitazone, approved by the US Food and Drug Administration in 1999 and European Medicines Agency in 2000 after several short-term phase I–III clinical trials, showed improvement in surrogates of blood glucose and HbA1c.^8^ However, meta-analyses of randomised trials published some 10 years later plus the large Rosiglitazone Evaluated for Cardiac Outcomes and Regulation of Glycaemia in Diabetes (RECORD) trial (4447 type 2 diabetes patients, 6 years follow-up) with the primary outcome cardiovascular hospitalisation or cardiovascular death, showed that the addition of rosiglitazone to standard care did not improve cardiovascular risk, and was associated with increased heart failure hospitalisation and myocardial infarction.^8^ Following reassessment, rosiglitazone was withdrawn from the market in September 2010. Second, trials of surrogate primary outcomes trials have been shown to overestimate the treatment effects by >40% (adjusted ratio of ORs: 1.46, 95% CI: 1.05 to 2.04), compared with trials using PRFOs.^9^ Such treatment effect overestimation can have fundamental implications for payer/reimbursement organisations such as the National Institute for Health and Care Excellence and funding and introduction of new therapies into healthcare systems that are not truly cost-effective.^10^

It would be expected that trials using a surrogate as primary outcome pay close attention to this aspect of design in their reporting, for example, clearly stating the outcome is a surrogate, providing a rationale for its use, and evidence of causality and validity (eg, meta-analysis of randomised trials demonstrating a strong association of the treatment effect on the surrogate and PRFO).^11^ However, this appears not to be the case; the most recent analysis, a review of randomised trials published in 2005 and 2006 found that 17% (107/626) used a surrogate primary endpoint and of these, only a third discussed whether the surrogate was validated.^12^

**Figure 1:**
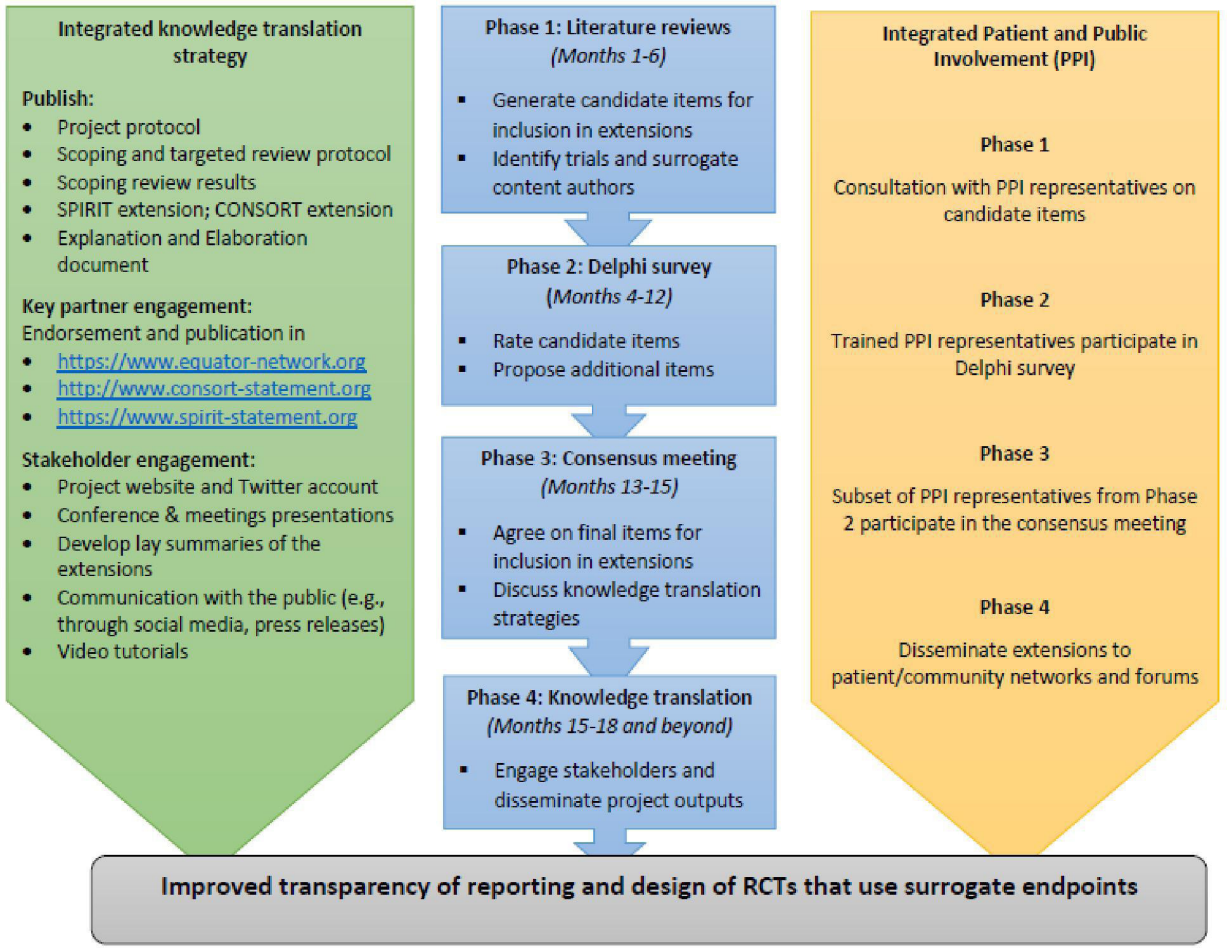
SPIRIT|CONSORT-SURROGATE extensions development steps. RCT, randomised controlled trial.

To address this challenge, SPIRIT|CONSORT-SURROGATE aims to develop extensions to the Standard Protocol Items: Recommendations for Interventional Trials (SPIRIT) 2013^13^ and Consolidated Standards of Reporting Trials (CONSORT) 2010 statements^14^ using the Enhancing Quality and Transparency of Health Research methodology (see figure 1).^15^ Interested stakeholders (trial methodologists, journal editors, healthcare industry, regulators and payers, and patient/public representative groups), particularly with interest/experience in the use of surrogates in trials, are invited to register their interest in taking part in the Delphi Survey process via the project website.^16^
